# Innate Immunity in Human Embryonic Stem Cells: Comparison with Adult Human Endothelial Cells

**DOI:** 10.1371/journal.pone.0010501

**Published:** 2010-05-05

**Authors:** Gábor Földes, Alexander Liu, Rekha Badiger, Mark Paul-Clark, Laura Moreno, Zsuzsanna Lendvai, Jamie S. Wright, Nadire N. Ali, Sian E. Harding, Jane A. Mitchell

**Affiliations:** 1 National Heart and Lung Institute, Imperial College, London, United Kingdom; 2 Heart Center, Semmelweis University, Budapest, Hungary; University of Tor Vergata, Italy

## Abstract

Treatment of human disease with human embryonic stem cell (hESC)-derived cells is now close to reality, but little is known of their responses to physiological and pathological insult. The ability of cells to respond via activation of Toll like receptors (TLR) is critical in innate immune sensing in most tissues, but also extends to more general danger sensing, e.g. of oxidative stress, in cardiomyocytes. We used biomarker release and gene-array analysis to compare responses in hESC before and after differentiation, and to those in primary human endothelial cells. The presence of cardiomyocytes and endothelial cells was confirmed in differentiated cultures by immunostaining, FACS-sorting and, for cardiomyocytes, beating activity. Undifferentiated hESC did not respond with CXCL8 release to Gram positive or Gram negative bacteria, or a range of PAMPs (pathogen associated molecular patterns) for TLRs 1-9 (apart from flagellin, an activator of TLR5). Surprisingly, lack of TLR-dependent responses was maintained over 4 months of differentiation of hESC, in cultures which included cardiomyocytes and endothelial cells. In contrast, primary cultures of human aortic endothelial cells (HAEC) demonstrated responses to a broad range of PAMPs. Expression of downstream TLR signalling pathways was demonstrated in hESC, and IL-1β, TNFα and INFγ, which bypass the TLRs, stimulated CXCL8 release. NFκB pathway expression was also present in hESC and NFκB was able to translocate to the nucleus. Low expression levels of TLRs were detected in hESC, especially TLRs 1 and 4, explaining the lack of response of hESC to the main TLR signals. TLR5 levels were similar between differentiated hESC and HAEC, and siRNA knockdown of TLR5 abolished the response to flagellin. These findings have potential implications for survival and function of grafted hESC-derived cells.

## Introduction

Human embryonic stem cells (hESC) are currently being developed as sources of tissue-specific cells for the treatment of human disease, including heart failure. It is hoped that hESC-derived cells can re-seed and repair damaged tissues allowing recovery of organ function. Although the immune response of the host to implanted cells has been the subject of a much interest, little is known about the innate immune response of the grafted cells themselves. Many cells of the body express a full or partial innate immune response, and these include both the endothelial cells [1] and cardiomyocytes [2], [3] which will be required to make a viable cardiac graft. The innate immune response is often modeled experimentally by activation of cells with pathogens or pathogen associated molecular patterns (PAMPs). The best studied of the PAMPs is lipopolysaccharide (LPS) derived from Gram negative bacteria. PAMPs are sensed by cells via pattern recognition receptors (PRRS), which include Toll like receptors (TLRs) [4]. LPS activates TLR4 which recruits adapter protein pathways including MyD88, MAL, TRIF and TRAM to initiate signaling events leading to activation of NFκB and the induction of inflammatory genes including CXCL8. There are 10 TLRs expressed in human cells with specific PAMPs for 1–9 identified. TLR10 remains an orphan receptor at present. In addition to the sensing of pathogens PRRs are now understood to sense host ligands as part of a wider role in the surveillance of danger signals [5]. Where phenotypes of TLR knock-out mice have been studied directly, TLR4 gene deletion, for example, is associated with immune suppression, chronic inflammation of the lung [6], vascular compromise and evidence of heart failure [7]. On the other hand, TLR activation in the heart is involved in the deleterious responses to oxidative stress [8], ischemia [9] and septic cardiomyopathies [10], and various TLR knockout mice are more resistant to these insults as well as to doxorubicin cardiomyopathy [11] and hypertrophy [12]. HESC originate from the inner cell mass of the blastocyst, which if left undisturbed would develop in the sterile environment of the womb. TLR responses in the embryo develop relatively late, and are again suppressed in the neonatal period [13]. The question of whether hESC and their derivatives in culture show a similar trend i.e. whether they retain the immature phenotype or develop the mature TLR responses, is therefore a vital question to understand in order to establish their potential in repair of the heart and other organs. In this paper we directly compare the expression and activity of TLRs, their downstream signaling components and NFκB signaling between undifferentiated and differentiated human hESC as well as with fully differentiated mature human aortic endothelial cells.

## Materials and Methods

### Materials

Heat inactivated *E. coli* and *S. aureus* were prepared as described previously [14]. Synthetic agonists for TLR1/2 (Pam_3_CSK_4_), TLR2/6 (FSL-1), TLR3 [poly(I:C)], TLR5 (Flagellin), TLR7 (Imiquimod), TLR8 (*E. coli* K12 ssRNA) were purchased from InvivoGen Co. (San Diego, USA). TLR4 ligand (LPS) was obtained from Sigma-Aldrich (Dorset, UK). IL-1 was purchased from R&D Systems (Abingdon, UK). All other reagents, unless otherwise stated, were obtained from Invitrogen.

### Human Embryonic Stem Cell Culture

Most of the experiments were performed using the H7 line from Geron Corporation© (Menlo Park, CA, USA). However, gene array analysis for undifferentiated stem cells was performed in H7, SHEF2, SHEF4 and SHEF5 lines. H7 and SHEF lines are ethically derived hESC lines. H7 was imported under a collaboration agreement with Geron, and with permission from the UK Stem Cell Bank.

Undifferentiated H7 cells were maintained under feeder-cell free conditions in mouse embryonic fibroblast-conditioned medium (MEF-CM), supplemented with 8 ng/ml of recombinant human basic fibroblast growth factor as per Geron's protocols described previously [15]. In brief, mouse embryonic fibroblasts (MEFs) were obtained from pregnant mouse embryos of MF-1 strain. After propagation in 10% FCS-containing medium, MEFs were mitotically inactivated with 0.01 mg/ml mitomycin C at passage 4 for 2.5 hours and adhered overnight onto pre-gelatinized T225 flasks (at a seeding density of 1.88×10^7^ cells/flask) in medium containing 10% FCS which was subsequently replaced with 150 ml of hESC medium, supplemented with recombinant human 4 ng/ml basic fibroblast growth factor. Conditioned medium, collected daily for up to 10 days, was passed through 0.2 µm low protein attachment cellulose acetate filter units (Corning) prior to feeding H7 cells. The hESC medium consisted of KnockOut DMEM (KO DMEM) supplemented with 20% KnockOut serum replacement (KOSR), 1 mM L-glutamine, 50 U/ml penicillin, 50 µg/ml streptomycin, 1% non-essential amino acids (100x stock) and 0.1 mM β-mercaptoethanol. All undifferentiated cells were cultured on Matrigel (BD Sciences)-coated 6-well plates (Nunc, Roskilde, Denmark). Before induction of differentiation, spontaneously differentiated cells were removed by treatment with collagenase at 37°C for up to 10 min. hESC colonies were mechanically broken with a 5 ml pipette tip and were cultured for 4 days in low attachment 6-well plates (Nunc), suspended in differentiation medium to form embryoid bodies. The differentiation medium was the same as the hESC medium except that the KOSR was replaced with 20% non-heat-inactivated FCS. Embryoid bodies were plated out onto 0.5% gelatinized dishes and cultured in order to allow continued differentiation.

### Cell Plating and Handling

Undifferentiated H7 cell colonies from 6-well plates were removed and subcultured in 96-well plates previously coated with Matrigel (100 ul/well). For this, medium was aspirated and spontaneously differentiating cells among colonies were removed by treatment with collagenase at 37°C for up to 10 minutes, after which time collagenase was aspirated and cells in each well were washed with 2 ml PBS. Colonies of undifferentiated H7 cells were broken up mechanically with a 5-ml pipette tip and small clusters were subcultured to >70% confluence. Differentiated hESC in T175 flasks or 10-cm culture dishes were removed from the surface by treatment with Trypsin-EDTA (Sigma-Aldrich), counted and plated onto 96 well plates coated with 0.5% gelatin.

### hESC-Derived Endothelial Cell (hESC-EC) Culture

Undifferentiated H7 hESC were dissociated into clumps and placed into ultra low-attachment plates in medium containing 2% FCS (Endothelial Growth Medium-2, Lonza). As described elsewhere [16], CD31+ cells were sorted by a sterile cell sorter (BD FACSAria, BD Biosciences) from cultures 13 days after differentiation and propagated in endothelial growth medium. Passages between 3 and 10 were used for experiments.

### Matrigel Tubule-Forming Assay

Matrigel was diluted 1∶2 with endothelial basal medium on ice and then 100 µl/well added to 24-well plates and allowed to gel in a thin layer at 37°C. CD31+ cells (50.000 per well) were seeded onto the gels and tubes were photographed after 22 hours.

### Endothelial Cell Culture

Primary human aortic endothelial cells were purchased from Promocell (Heidelberg, Germany) and cultured according to manufacturer's instructions. The human endothelial cell line (EAhy-926) was cultured in Dulbecco's Modified Eagle's Medium (DMEM) supplemented with 1% Hypoxanthine-Aminopterin-Thymidine (all Sigma-Aldrich), 5 mM L-glutamine, 100 U/ml penicillin, 100 µg/ml streptomycin, and 10% heat-inactivated FCS.

### Cell Treatments

To investigate the response to TLR stimulation, cells were treated in 96 well plates with PAMPS which are agonists to TLR1-8, or heat killed *S. aureus* and *E. coli* while IL-1 was used as a positive control, for 24 hours. For experiments to measure NFκB activation cells were treated for 1 hour.

### Knockdown of TLR5

For TLR5 siRNA knockdown, ON-TARGETplus SMARTpool TLR5 siRNA transfection was performed using DharmaFect reagent (100 nM, final incubation volume 100 µl) per manufacturer's instructions (Dharmacon, Thermo Scientific). Scrambled, non-targeting siRNA (100 nM; Dharmacon, Thermo Scientific) was used as negative controls. Fluorescent siGLO Red siRNA indicator (100 nM; Dharmacon, Thermo Scientific) was used for optimization and documentation of transfection efficacy (>90%) after 24–48 hours.

### Immunocytochemistry

Cell were fixed with 4% paraformaldehyde, permeabilized with 0.2% Triton X-100, and labeled with primary antibodies anti-CD31 (PECAM-1, 1∶100 dilution, Santa Cruz or Biolegend), anti-CD34 (Abcam, 1∶200), anti-myosin heavy chain (Ab15, 1∶200, Abcam, Cambridge), anti-von Willebrand factor (1∶100, Dako). Primary antibodies were detected with Alexa 488- (Invitrogen) and Alexa 647- (Invitrogen) conjugated secondary antibodies (all 1∶400). DNA was visualised with DAPI (0.5 µg/ml; Sigma). DiI-labelled acetylated human low density lipoprotein (Ac-LDL) was purchased from Invitrogen. Images were acquired on Zeiss Axio Observer Z1 fluorescence microscopy.

### Enzyme-Linked-ImmunoSorbent-Assays (ELISAs)

Concentrations of CXCL8/IL8 in cell-free supernatants were measured using sandwich ELISA kits (R&D Systems) and calculated using 4-parameter-log-fit curves according to manufacturer's protocols.

### Measurement of NFκB activation

Undifferentiated hESC were cultured in 6-well plates until 90% of the well surfaces were covered with H7 colonies before they were incubated with IL-1β (1 ng/ml) and LPS (1 µg/ml). After one hour, the cellular nuclear extracts were prepared using a commercially available nuclear extraction kit (Active Motif, Carlsbad, CA, USA) according to the manufacturer's protocols. In brief, the cells were washed, collected in ice-cold PBS in the presence of phosphate inhibitors and centrifuged at 500×g for 5 min. The resultant pellets were re-suspended in excess hypotonic buffer, treated with detergent and centrifuged briefly at 14,000×g for 30 sec. After the cytoplasmic fraction was collected, the nuclei were lysed and nuclear proteins were dissolved in a cocktail of lysis buffer and protease inhibitor. Nuclear protein concentrations were determined using the Bradford assay before subsequently analyzed for NFκB activation using the TransAM™ NFκB p65 transcription factor assay kit (Active Motif), according to the manufacturer's instructions. This assay is based on nuclear NF-κB p65 proteins binding to consensus NF-κB oligonucleotides fixed in 96-well plates. In brief, 10 µg of nuclear proteins were added to each well and incubated for 1 h to allow the binding of P65 to consensus oligonucleotides. The presence of the resulting complex was detected by a primary antibody. After the addition of a horseradish peroxidase conjugated secondary antibody, P65 was quantified by spectrophotometry.

### Gene-Expression Analysis

#### RNA Extraction

Total RNA was isolated from hESC using the RNeasy kit (Qiagen, Hilden, Germany) according to manufacturer's protocols. Spin-column samples were centrifuged at 9500×g, 20°C for 15 seconds unless stated otherwise. In brief, 350–600 µl of lysis-Buffer RLT was added to cell-pellets and homogenized by passing through a 20-gauge needle 5–10 times before the addition of equal volume of 70% ethanol. The solution was centrifuged in a spin-column before DNase digestion using DNase-Buffer RDD solution (Qiagen) at room temperature for 25 minutes. Subsequently, RNA was washed with Buffer RW1 and RPE under centrifugation. A final wash with Buffer RPE for 2 minutes was applied before the RNA was eluted in 30–60 µl of RNase-free water. RNA concentration and integrity was determined by spectrophotometry (Spectramax, Switzerland) to obtain A_260_/A_280_ ratios and later by agarose gel electrophoresis.

#### Reverse transcriptase-PCR First-Strand reaction

The first-strand-complimentary-DNA (cDNA) was synthesized from 1 µg of total RNA using the RT^2^ First-Strand Kit (SuperArray Bioscience Corporation, MD, USA) according to manufacturer's protocols. Briefly, 10 µl of rt-Cocktail, containing RNA was mixed with 10 µl Genomic-DNA-Elimination Mixture and incubated for 15 minutes at 42°C then for 5 minutes at 95°C to inactivate/degrade RNA and reverse transcriptase enzyme. 91 µl of RNase-Free water was added to the remaining cDNA.

#### Real Time-Polymerase Chain Reaction (RT-PCR) Array

RT-PCR preparations were performed with RT^2^-Profiler-PCR-Array kit (SuperArray) according to manufacturer's protocols. In brief, the experimental cocktail was made up containing 102 µl diluted first-strand cDNA, 1.275 ml SuperArray-RT^2^-SYBR-Green/ROX-MasterMix and 1.173 ml RNase-Free water. 25 µl of cocktail was added into each well of the 96-well plate, containing forward and reverse primers for TLR-related genes, housekeeping genes, human-genomic-DNA-contamination and PCR controls. Samples were amplified using ABI 7500 Real-Time-PCR System (Applied Biosystems, Foster City, USA) for 40 cycles of 15 s at 95°C, 30 s at 55°C, and 30 s at 72°C. Relative levels of gene expression (fold differences) were calculated according to manufactures instructions. C_T_ values which were at 35 or higher were considered as indicating undetectable levels of expression. If 3/3 or 2/3 runs produced C_T_> = 35 for a given gene, the expression was considered undetectable overall.

#### Quantitative RT-PCR for TLR5

For quantifying mRNA levels of TLR5 in undifferentiated and differentiated hESC cultures, real-time PCR analyses were performed with TaqMan Gene Expression Assay (Hs01920773_s1, Applied Biosystems, CA). Human GAPDH Endogenous Control (FAM/MGB probe) was used as a housekeeping control. The PCR was performed with Rotor-Gene 3000 (Corbett Research) real-time PCR instrument and the relative expression was determined.

### Statistical analysis

Data is reported as the mean± S.E. mean for n experiments. Data was analyzed using one-way ANOVA followed by Dunnett's Multiple Comparison Test or by one-sample t-test for normalized data as described in the respective legends.

## Results

### Phenotype of human hESC differentiated to include endothelial-like phenotypes

**Figure 1:**
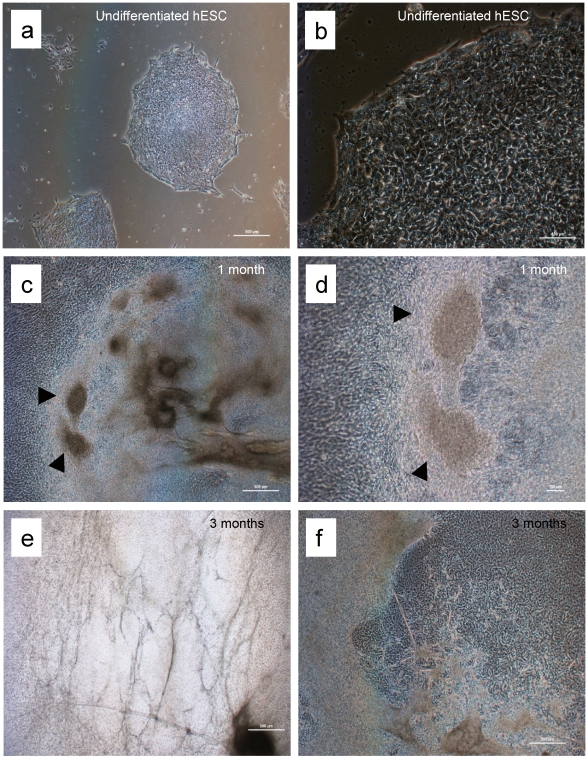
Appearance of hESC cultures. Undifferentiated H7 cells (a, b); after 1 month (c, d) and 3 months (e, f) of differentiation. Examples of clusters of beating cells are seen in c and d, and are shown in the Video S1. Vessel-like structures can be observed (e).

**Figure 2:**
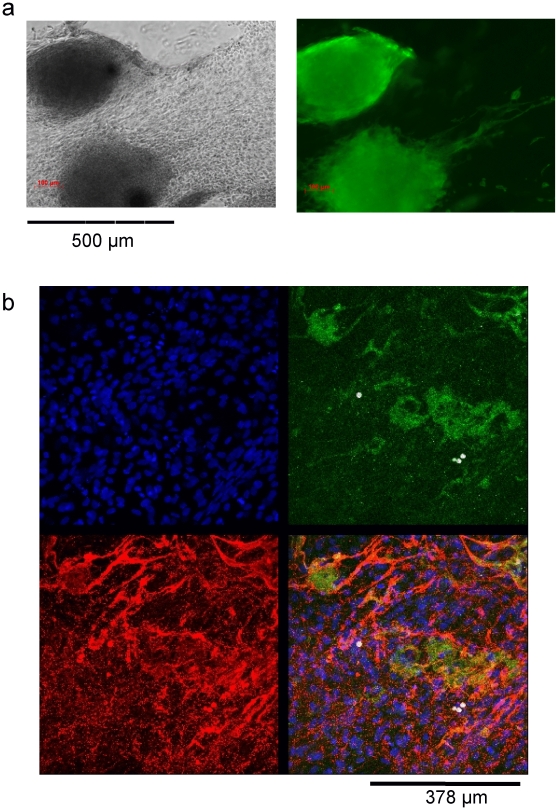
Presence of cardiomyocytes and endothelial cells in differentiated hESC cultures. Immunocytochemical staining of clusters of 1 month differentiated H7 hESC showing (a) cardiomyocytes myosin heavy chain (α, β) (green) with corresponding brightfield image and (b) endothelial cells identified with von Willebrand factor (green), CD31 (red) and DAPI (nuclei, blue).

Undifferentiated hESC have a characteristic appearance as tightly packed cells in colonies as shown in Fig. 1a and b. The H7 line, obtained from Geron Corp., Menlo Park CA, was grown under feeder-free conditions as described previously [15] and differentiated via embryoid body formation in 20% FCS. After four days of differentiation in suspension cultures, embryoid bodies were plated out onto gelatinized surfaces and continued to differentiate in adherent cultures for prolonged periods (over 4 months). Figure 1C-F shows the morphology of cultures at 1 and 3 months after differentiation, demonstrating the emergence of a variety of features, including clusters of beating cardiomyocytes (video S1) and vessel-like structures. Immunocytochemical staining for known markers (Fig. 2) confirms the presence of cardiomyocytes and endothelial cells within the mixed population of cells.

**Figure 3:**
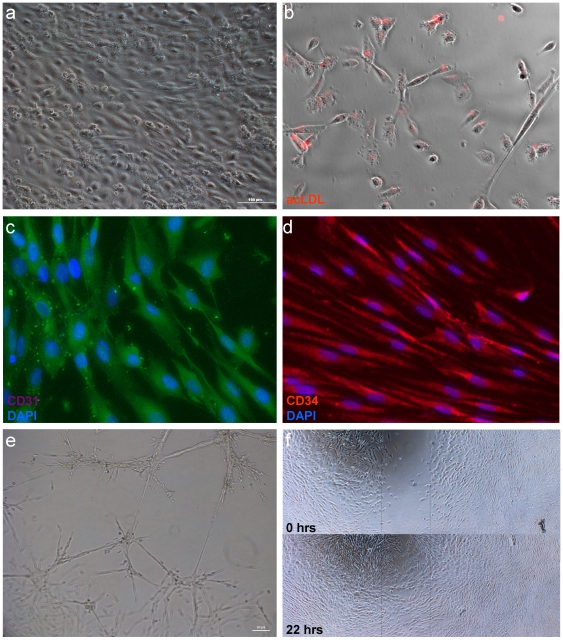
Characteristics of human embryonic stem cell-derived endothelial cells. The hESC-EC cultures showed (A) cobblestone morphology. (B) Cells showed DiI-labelled acetylated LDL uptake, and were stained positive for human anti-CD31 antibody (green; C), and anti-CD34 (red; D). DAPI (blue) was used for nuclear staining. (E) Cells plated on solidified Matrigel form hollow-like tubes. (F) In a wound healing assay, cells show migration on fibronectin surface (upper panel showing cell free area at 0 hours; cells migrate into the wound site at 22 hours).

By adjustment of differentiation conditions and using FACS sorting for CD31 surface antigen, a highly expandable population of human endothelial-like cells (hESC-EC) was obtained from the hESC. Cells took on a cobblestone pattern in culture characteristic of endothelial cells (Fig. 3A). Cells were stained positive for endothelial-specific CD31 and CD34 markers (Fig. 3C–D) and acetylated LDL uptake in culture (Fig. 3B). Further indicating their endothelial phenotype and function, cells formed tube-like structures on solidified Matrigel and showed migration on fibronectin surface in wound healing assays (Fig. 3E–F).

### Relative expression of TLRs and related genes in hESC and in endothelial cells

**Figure 4:**
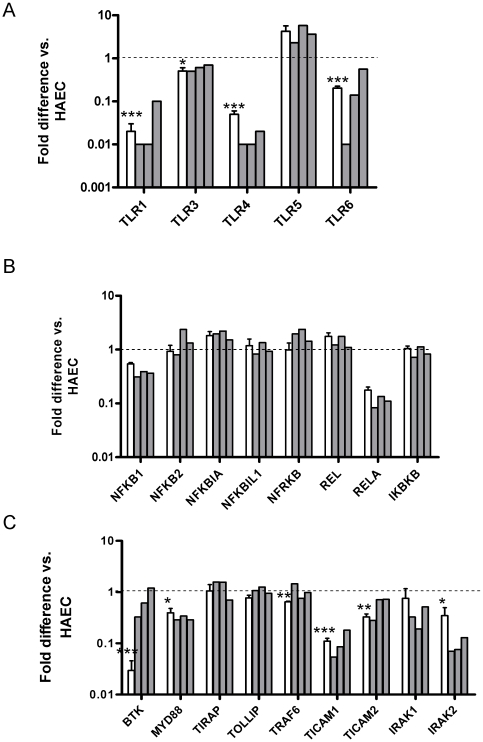
Expression of TLR and TOLL or NFκB signalling genes in undifferentiated hESC. Relative expression levels in undifferentiated H7 hESC (n = 3, open bars) and each of 3 SHEF lines (grey bars) compared to human aortic endothelial cells (HAEC, n = 3). A: TLR genes. TLRs 2 and 7–10 were undetectable in the majority of HAEC. B: NFκB related genes; C: TOLL signalling-related genes. Statistical significance was calculated using one-sample t-test for averaged H7 values against 1.0, *P<0.05, **P<0.01, ***P<0.001, n = 3.

Expression of TLRs and downstream signaling effector genes was determined in undifferentiated hESC as well as 1–4 months after differentiation. Fig. 4A shows expression levels of TLRs 1, 3, 4, 5 and 6 in undifferentiated H7 cells and in each of 3 SHEF lines. TLR 1, 3, 4 and 6 expression was consistently lower in hESC compared to endothelial cells, with TLRs 1 and 4 particularly low. Levels of TLR8, and TLR10 were undetectable in hESC (Cycle threshold, C_T_, values of 35 or lower in the majority of samples (Table S1)). Levels of TLR2 were similar to or higher than those of TLR6 in both H7 and SHEF lines, with C_T_ values in the range 27–30 (Table S1), but as levels were undetectable in endothelial cells in the majority of samples a fold-change was not calculated. Low levels of expression of TLR7 and TLR9 were also present in hESC but undetectable in endothelial cells. Interestingly, expression of TLR5 was robust in endothelial cells but even higher in undifferentiated hESC. Agreement was good between H7 and SHEF lines for TLR expression. Consistent with the activity of the downstream signalling pathways, the majority of both NFκB (Fig. 4B) and TLR signaling genes (Fig. 4C) were expressed at similar levels between endothelial cells and hESC, though reduced expression of MyD88 and TICAM1 might also contribute to the poor functional response. Once again, there was reasonable agreement between H7 and SHEF lines.

### Relative expression of TLRs and related genes following differentiation of hESC

**Figure 5:**
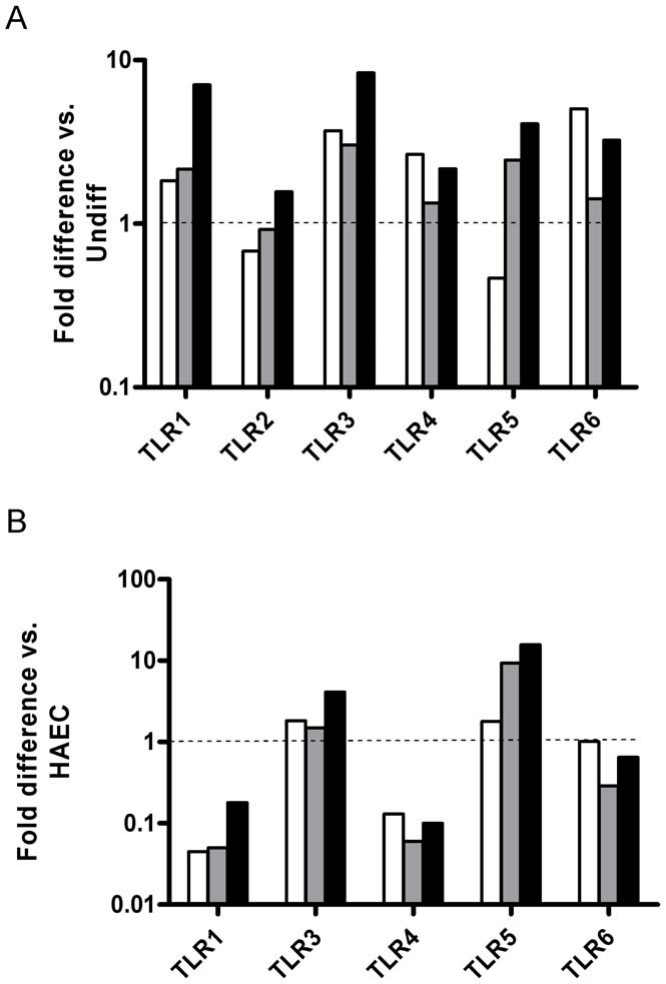
Expression of TLR genes in differentiated hESC. Relative expression in differentiated H7 hESC at 1 month (n = 2, open bar), 3 months (grey bar) and 4 months (solid bar) compared to A: averaged undifferentiated H7 hESC (n = 3) and B: human aortic endothelial cells (HAEC, n = 3).

Expression levels were determined at 1, 3 and 4 months after differentiation of H7 hESC. Compared to undifferentiated H7 (Fig. 5A), there was a general increase with time in TLRs 1, 2, 3, 4, 5 and 6, with all above undifferentiated levels by 4 months. Low levels of TLRs 7, 9 and 10 also became sporadically apparent (Table S1). However, comparing differentiated hESC with endothelial cells (Fig. 5B) it is clear that TLR1 and TLR4 were still at low levels, even after 4 months of differentiation. Only modest adjustments of expression level of the NFκB (Table 1) levels were seen during differentiation while some components of the TLR signaling pathways (TICAM1, TICAM2 and IRAK1) were consistently increased (Table 2). A full list of gene expression changes during differentiation is found in Table S2.

**Table 1:** NFκB related gene expression in hESC.

Table 1	Differentiated: fold change vs undifferentiated			
Gene name	1 month	1 month	3 month	4 month
NFKB1	1.66	1.92	0.38	0.85
NFKB2	2.41	2.28	0.63	2.01
NFKBIA	1.12	0.68	0.26	1.09
NFKBIL1	1.21	0.91	0.47	1.28
NFRKB	1.61	0.83	0.28	0.91
c-REL	0.75	0.41	0.12	0.76
NFKB3	6.61	2.98	0.86	2.05
IKBKB	0.77	0.41	0.31	0.60

Fold change of differentiated H7 hESC at 1–4 months compared to average (n = 3) undifferentiated H7, where a value of 1 represents equivalent gene expression and of less than 1 represents lower expression in differentiated than undifferentiated.

**Table 2:** TOLL signalling related gene expression in hESC.

Table 2	Differentiated: fold change vs undifferentiated			
Gene Name	1 month	1 month	3 month	4 month
BTK	n.d.d	n.d.d	n.d.d	n.d.d
MYD88	3.82	0.89	0.47	0.89
TICAM2	2.84	3.23	1.18	3.10
TIRAP	1.56	1.12	0.37	0.87
TOLLIP	1.42	1.62	1.74	1.14
TRAF6	2.08	1.76	1.00	1.41
TICAM1	3.65	5.53	3.12	3.80
IRAK1	1.34	4.23	1.50	1.84
IRAK2	0.75	0.66	0.26	1.01

Fold change of differentiated H7 hESC at 1–4 months compared to average (n = 3) undifferentiated H7, where a value of 1 represents equivalent gene expression and of less than 1 represents lower expression in differentiated than undifferentiated. N.d.d  =  not detectable in differentiated cells.

### Effect of PAMPs for TLR1-8 and whole bacteria on CXCL8 release by human hESC and primary human endothelial cells

**Figure 6:**
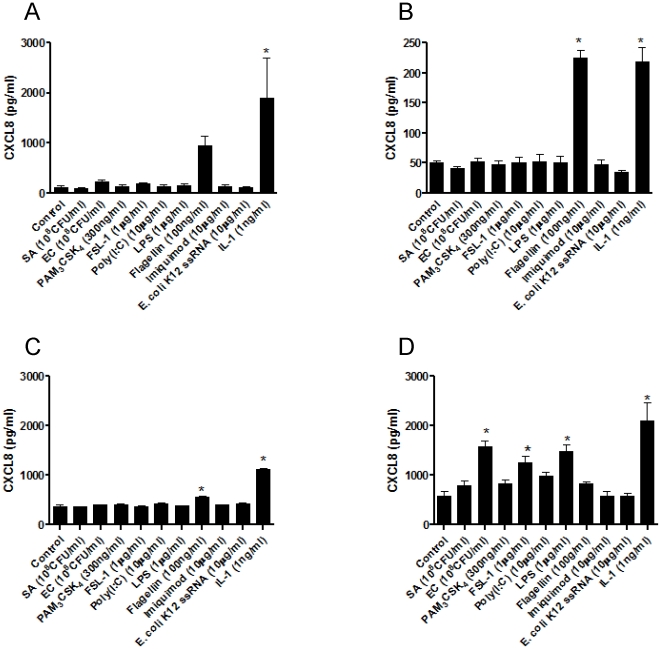
Response of hESC and primary cells to bacteria and PAMPs. Release of CXCL8 from undifferentiated (A), 1 month- (B) or 4 month- (C) differentiated H7 hESC or from primary cultures of human aortic endothelial cells (D). Cells were treated with Gram negative E. coli (C, 10^8^ CFU/ml), Gram positive S. aureus (SA, 10^8^ CFU/ml), PAMPs for TLR2/1 (PAM_3_CSK4; 300 ng/ml), TLR2/6 (FSL-1; 1 mg/ml), TLR3 (Poly:IC; 10 mg/ml), TLR4 (LPS; 1 mg/ml), TLR5 (flagellin; 100 ng/ml), TLR7 (imiquimod; 10 mg/ml) or TLR8 (E. coli 12 ssRNA; 10 mg/ml) or IL-1β (IL-1; 1 ng/ml) for 24 hours before CXCL8 was measured using ELISA. The data are the mean ± S.E. mean for n = 8−11 (A); n = 3−9 (B); n = 3 (C) and n = 4−6 (D). Statistical significance was calculated by one-way analysis of variance followed by Dunnett's Multiple Comparison Test (to control). A p value of less than 0.05 was considered statistically significant and denoted by *.

**Figure 7:**
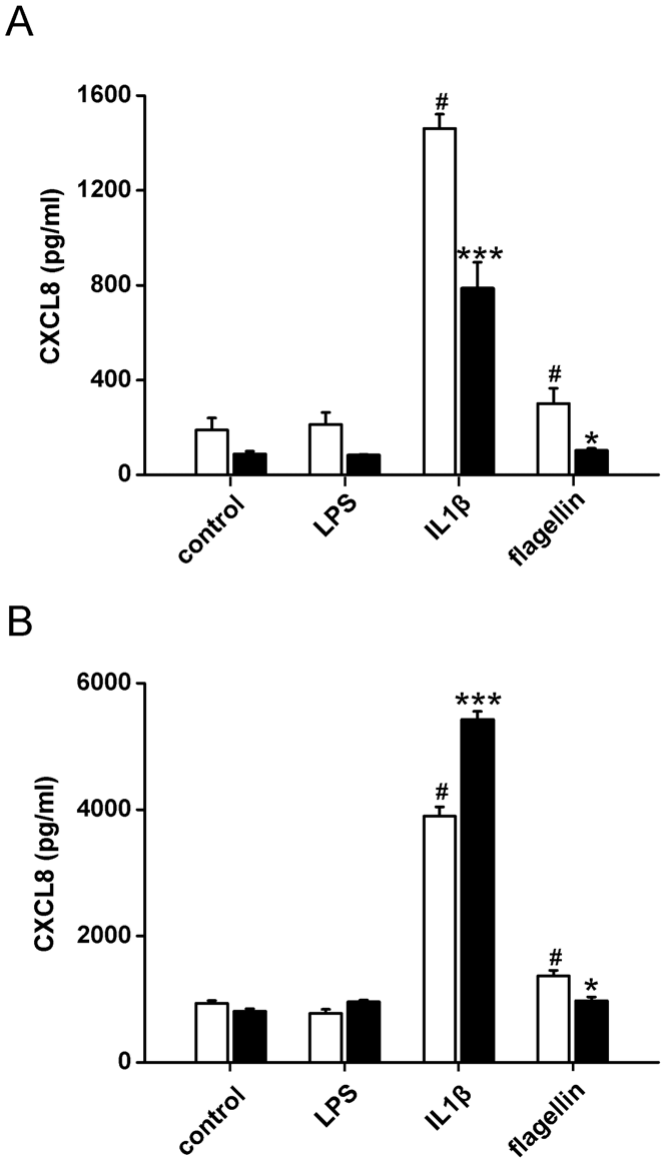
Enzyme-linked immunosorbent assay of CXCL8 production by hESC and purified hESC-derived endothelial cells. Bar graphs showing release of CXCL8 from 1 month-old differentiated H7 hESC cells (A) or hESC-derived CD31^+^ endothelial cells (B) transfected with scrambled siRNA (open bars) or TLR5 siRNA (solid bars) for 48 hours. Transfected cells were treated with TLR4 (LPS; 1 mg/ml), IL-1b (IL-1β; 1 ng/ml), or TLR5 (flagellin; 100 ng/ml) for 24 hours before CXCL8 was measured. The data are the mean ± S.E. mean for n = 3. # P<0.05 vs control; * P<0.05 and *** P<0.001 vs scrambled siRNA group.

Release of CXCL8 was used in our study as a biomarker of cell activation. In undifferentiated hESC there was no statistically significant increase in CXCL8 release in cells stimulated for 24 h with whole *E. coli*, *S.Aureus* or with an array of PAMPs for TLRs1, 2, 3, 4, 6, 7 or 8 (Fig. 6A). This was then compared with differentiated hESC cultures, in which phenotypic evidence of ESC specialization had been established (Fig. 2). One month after initiation of differentiation, hESC-derived cells still did not release increased levels of CXCL8 in response to PAMPs for TLRs or whole bacteria (Fig. 6B). The exception was flagellin, an activator of TLR5, which produced an increase in CXCL8 in both undifferentiated hESC and differentiated hESC-derived cells (Fig. 6A and B). Remarkably, even by 4 months of differentiation, TLR5 remained the only PAMP to produce a response in hESC-derived cells (Fig. 6C). IL-1β, which acts independently of TLRs but via the TLR adapter protein MyD88, activated undifferentiated or differentiated hESC to release CXCL8 (Fig. 6A–C). In separate experiments protocols were repeated to compare directly responses in purified cultures of hESC-EC and mixed differentiated hESC cultures (Fig. 7A–B). Similar results were seen to those presented in Fig. 6. LPS had no effect on CXCL8 release by mixed differentiated cultures of hESCs (Fig. 7A) or purified hESC-ECs (Fig. 7B). Again flagellin and IL-1b increased CXCL-8 release in these experiments (Fig. 7A–B). Purified hESC-EC also expressed TLR5, showing a 3-fold increase in mRNA levels as compared with those in undifferentiated hESC (data not shown). By contrast to results obtained with hESC and their differentiated derivatives, primary cultures of adult human aortic endothelial cells released increased levels of CXCL8 in response to Gram negative bacteria and LPS (which activate TLR4), FSL-1 (which activates the TLR2/6 heterodimer) or IL-1β (Fig. 6D). Similar results were seen when the endothelial cell line, EAhy-926, was used (data not shown). To ensure that differences in CXCL8 release between hESC and primary endothelial cells were not due to the different media used for routine culture, both undifferentiated and differentiated hESC were re-tested in the DMEM plus 10% FCS used for EAhy-926 endothelial cell culture (Fig. S1). Use of the EAhy-926 media altered basal CXCL8 levels to some extent but did not reveal an effect of bacterial or PAMP stimulation on CXCL8 release. However, it should be noted that the effects of long term culture in highly specialized medium may well influence TLR and other signaling pathways in cells.

### Knockdown of TLR5 confirms specificity of flagellin response

Silencing of TLR5 using siRNA resulted in a significantly reduced CXCL8 release in flagellin-treated mixed differentiated hESC cultures (Fig. 7A) or in purified hESC-EC (Fig. 7B) compared to scrambled siRNA. Interestingly, TLR5 siRNA knockdown had a general depressant effect on CXCL8 release in mixed differentiated cultures, while the reduction was specific to flagellin in the hESC-EC.

### Effect of cytokines and the TLR4 PAMP LPS on cell and NFκB activation in hESC

**Figure 8:**
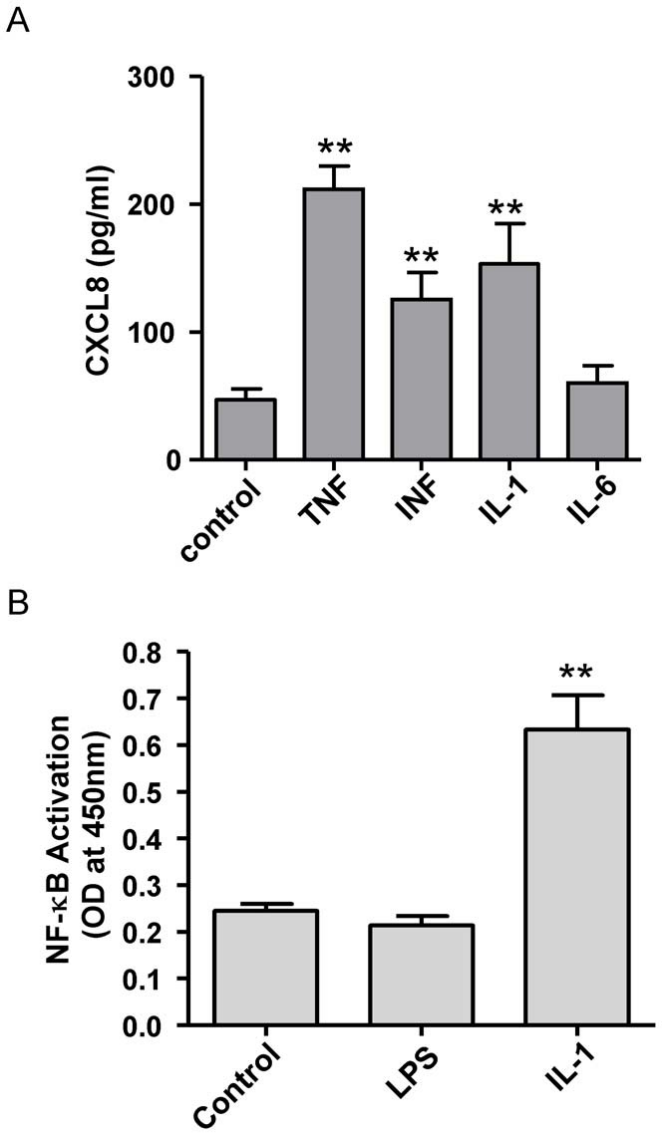
Activation of NFκB in undifferentiated hESC. (A), Undifferentiated H7 hESC were activated to release CXCL8 after 24 hours stimulation with TNFα (TNF), INFγ (INF) or IL-1β (IL-1) but not by IL-6 (10 ng/ml for each)(n = 6). (B), NFκB was activated in undifferentiated H7 hESC following 1 hour stimulation with IL-1β (1 ng/ml) but not by LPS (1 µg/ml) (n = 3). Statistical significance was calculated by one-way analysis of variance followed by Dunnett's Multiple Comparison Test (to control). **P<0.01.

To further investigate the underlying mechanism for the lack of TLR responses in hESC, the function of downstream pathways was investigated. Effects of IL-1β on CXCL8 release by undifferentiated hESC were compared with that produced by TNFα, INFγ and IL-6. It was found that cells were activated to release increased levels of CXCL8 by IL-1β, TNFα, INFγ but not by IL-6 (Fig. 8A). In line with observations above, NFκB was activated in undifferentiated hESCs following 1 h stimulation with IL-1β whilst treatment of cells with LPS for the same period had no effect (Fig. 8B). Clearly, as evidenced by our data, we confirm the results of others [17], [18], [19] that NFκB genes are functionally active in undifferentiated hESC but the TLR4 PAMP is unable to produce a NFκB translocation in these cells.

## Discussion

The primary findings of the present study are first, that undifferentiated hESC had no immune response associated with bacterial TLR activation, or a range of bacterial PAMPs except for TLR5. More surprisingly, even cultures of hESC differentiated for up to 4 months had no detectable immune response to TLR2 (Gram positive) or TLR4 (Gram negative) activation. The TLR4 ligand, LPS also failed to activate NFκB in hESCs, consistent with the notion that these cells do not have functionally active TLR responses. A population of differentiated hESCs enriched for endothelial-like cells (hESC-EC) shared the lack of response through TLR4 and active response through TLR5.

HESC were directly compared with endothelial cells cultured from adult tissue, and these were shown to display positive bacterial-TLR response as assessed by increased CXCL8 release when cells were stimulated with agonists of TLR2 or TLR4. Gene expression profiles in both undifferentiated and differentiated hESC revealed relatively low levels of TLR1 or TLR4 in hESC compared to levels expressed in endothelial cells. Moreover, TLR5 expression was relatively high in differentiated hESC compared to endothelial cells. Whilst such gene expression data should be interpreted with caution, this pattern of TLR expression fits well with the ability of hESC to respond to PAMPs selective for these TLRs. It should be noted that TLR2 acts as a dimer with either TLR1 or TLR6. Loss of TLR1 would therefore indirectly influence the activation through TLR2.

TLRs are linked via TIR domains to adapter protein pathways such as MyD88. Our findings show that hESC and purified hESC-EC responded to IL-1β, which is an agonist that works independently of TLRs but via a TIR (TLR IL1 receptor) domain linked receptor, by releasing increased levels of CXCL8 and activation of NFκB. HESCs were found to express a number of NFκB related genes. When levels were compared with those in endothelial cells the relative expression of NFκB2, NFκB1A, NFKNFRκB, REL and IKBκB were similar in hESC. However, expression levels of NFκB1 (gene for p50) or RELA (gene for p65) were lower in hESC than in endothelial cells. The lower gene expression clearly did not limit NFkB activation or CXCL8 induction. It was interesting to note that, in addition to IL-1β, hESCs responded by releasing increased levels of CXCL8 when stimulated with other cytokines including TNFα, INFγ, but not IL-6. These data also suggests that whilst hESC and hESC-EC are not able to sense and respond to bacterial PAMPs such as LPS, they clearly have intact active inflammatory signaling pathways. Gene expression analysis of these adapter proteins and related genes showed some differences between hESC and the adult endothelial cells. Whilst levels of TIRAP, TOLLIP and TRAF6 are similar in undifferentiated hESC and adult endothelial cells, the levels of BTK, MyD88, TICAM1, TICAM2 and IRAK2 were significantly lower. Interestingly the pattern of gene expression changed somewhat when hESC were differentiated, with upregulation of TICAM1, TICAM2 and IRAK1. Changes in expression of NFκB-related genes during differentiation were modest and not consistent, although RELA was upregulated while REL was reduced (Table 2). However, functional levels of MyD88 and associated signaling proteins were clearly sufficient in both differentiated and undifferentiated hESC to mount a robust inflammatory response to IL-1β.

NFκB signaling in differentiating hESC has been somewhat controversial, with a study on the hES-NCL1 line showing expression of NFκB and pathway components at significant levels in undifferentiated cells but down-regulating during differentiation and possibly controlling the differentiation process in this way [19]. In contrast, Kang et al [17] found very low NFκB in undifferentiated SNUhES3 and MizES4 hESC lines compared to HEK and haematopoetic progenitor lines (although MyD88 and TRAF2 were equivalent), as well as poor CXCL8 induction by TNFα. Differentiation was associated with increased NFκB and IL8 response to TNFα, a finding that was replicated in mouse ESC [18]. The contradictory results in these studies might suggest variation between hESC lines, but we have seen robust and similar expression levels of the key NFκB pathway components in various undifferentiated hESC (H7 and three of the SHEF lines), with little change upon differentiation in H7.

The lack of response to bacterial challenge may not be surprising for the undifferentiated hESC, given the delay in development of innate immune sensing in the embryo. Full TLR responses do not generally develop until near or even after full term. Our results are in agreement with others [20] who also reported lack of responsiveness of undifferentiated mouse ESC and their differentiated derivatives to LPS. These authors further reported that lack of TLR4 expression in these cells was due to epigenetic modulation of the TLR4 gene promoter (methylated). However, most recently a study by Lee and co-workers [21] demonstrate positive expression of TLRs in mouse ESCs. Moreover these authors demonstrated that after long term exposure (24 days) increased proliferation and differentiation of mouse ESCs was seen in cells stimulated with LPS (TLR4) or POLY:IC (TLR3). The apparently conflicting observations in mouse ESCs are likely influenced by different culture conditions, time of LPS exposure, epigenetic factors and differing clones of ESCs used. The likely similarities and differences between human and mouse ESC cells in this and other respects remain the subject of investigation. Clearly our data shows that hESC are resistant to stimulation with PAMPs except for flagellin. We found that hESC and purified hESC-EC expressed TLR5 and that knockdown of TLR5 by siRNA inhibited CXCL8 production in response to flagellin in both cultures. This suggests that TLR5 acts as an active sensor for bacterial flagellin monomers in hESC. Evidence for the cytoprotective role of TLR5 comes from studies showing that TLR5 ligation can block apoptosis by activating downstream antiapoptotic genes [22]. Activation of TLR5 may protect against tissue injury in conditions involving high levels of cell death [23].

The mechanism responsible for the lack of LPS responsiveness in hESC is still to be determined. However, it has been observed that mesoderm formation in embryoid bodies (EB) from hESC can be inhibited (as shown by mesoderm specific gene, Brachury silencing), by challenge with LPS, the TLR4 PAMP [24]. This suggests either that low TLR4 levels are able to produce a sufficient response to affect differentiation processes with prolonged stimulation, or that the results were secondary to LPS release of cytokines from the MEF layer present during EB formation in that study.

Lack of innate immune and danger-sensing signals has different implications for different cell lineages depending on whether, like endothelium, they have a distinct role against pathogens or, like cardiac myocytes, the pathways involved have been directed towards more general danger signals. Endothelial cells not only provide barrier and endocrine functions but are also essential innate immune surveillance cells. Indeed, endothelial cells are generally the first cell type that pathogens encounter in the circulation. For many target cells, including endothelial cells, it is essential that tissue derived from hESCs express a functional bacterial innate immune response. The use of hESC-derived endothelial cells for tissue repair may therefore be compromised by the lack of innate immune sensing. It remains to be seen whether the *in vivo* environment will stimulate maturation of the hESC-derived cells and, if not, what mix of host and grafted endothelium will be tolerable to maintain function. It may be necessary to use pre-treatment strategies to accelerate maturation *in vitro* such as mechanical or hormonal stimulation, provision of extracellular matrix or co-culture with other cell types. For cell types not directly involved in immune sensing, the consequences of relative insensitivity to insult may have both positive and negative aspects. Cell therapy will, in most cases, be directed to areas of damage and implanted hESC-derived cells will be introduced to areas of hypoxia or inflammation. Taking the example of cardiac myocytes, the lack of TLR2 and 4 response would be predicted to increase resistance to hypoxia [10] and so improve survival after implantation in scar border zone, although the sensitivity to the inflammatory cytokine milieu of the infarcted heart will be retained. This might suggest implantation success would be optimal at a later time period after infarction, when acute inflammation has subsided and scar is more established.

### Summary

In summary, we have shown for the first time that hESC do not sense or respond to bacteria or the bacterial PAMPs that activate TLR4, but do respond to flagellin which activates TLR5. We show that this pattern of PAMP sensing is consistent with the relative expression of TLR genes in hESCs. We show that despite having no ability to sense LPS, hESC respond in a robust manner to cytokines linked to MyD88 and NFκB transcription pathways. These observations are important as they suggest that whilst endothelial (and other) cells produced from hESC may display phenotypic markers they do not express a mature immune function. This has implications for the strategy and timing of implantation of hESC derived cells for tissue repair.

## Supporting Information

Figure S1 Comparison of hESC culture media. Either undifferentiated (A) or 1 month differentiated (B) hESC were tested either in their routine culture media (as detailed in the methods) (closed bars) or with 10% FCS-containing DMEM 24 hours before and during stimulation (open bars). PAMPs or IL-1β was added to cells for 24 hours before CXCL8 was measured by ELISA (n = 3). Statistical significance was calculated by one-way analysis of variance followed by Dunnett's Multiple Comparison Test (to control). **P<0.01.
(0.50 MB TIF)

Table S1 CT values for human aortic endothelial cells (HAEC, n = 3), undifferentiated H7 hESC (n = 3); undifferentiated SHEF2, SHEF4 and SHEF5 and differentiated H7 at 1 month (n = 2), 3 months and 4 months after differentiation. Values >35 were set to 35: these were considered as undetectable.
(0.36 MB DOC)

Table S2 Fold changes between undifferentiated H7 hESC (n = 3) and differentiated H7 at 1 month (n = 2), 3 months and 4 months after differentiation. N.d.d.  =  not detectable in differentiated; n.d.u.  =  not detectable in 3/3 or 2/3 undifferentiated and n.d.  =  not detectable in either.
(0.14 MB DOC)

Video S1 1 month differentiated H7 hESC showing clusters of beating cardiomyocytes, as shown in Fig. 1C and D.
(2.26 MB WMV)
