# Supplementary material for: Innate Immunity in Human Embryonic Stem Cells: Comparison with Adult Human Endothelial Cells
Source: PLoS One. 2010 May 5;5(5):e10501. doi: 10.1371/journal.pone.0010501 (PMC2864770; doi:10.1371/journal.pone.0010501)
Supplement: Table S1 — CT values for human aortic endothelial cells (HAEC, n = 3), undifferentiated H7 hESC (n = 3); undifferentiated SHEF2, SHEF4 and SHEF5 and differentiated H7 at 1 month (n = 2), 3 months and 4 months after differentiation. Values >35 were set to 35: these were considered as undetectable. (0.36 MB DOC) [file pone.0010501.s002.doc]

Table S1

|  |  |  |  |  |  |  |  |  |  |  |  |  |  |  |  |  |
| --- | --- | --- | --- | --- | --- | --- | --- | --- | --- | --- | --- | --- | --- | --- | --- | --- |
|  | **HAEC** | **HAEC** | **HAEC** |  | **H7** | **H7** | **H7** |  | **SHEF 2** | **SHEF 4** | **SHEF 5** |  | **H7** | **diff** |  |  |
| **Gene Name** | |  |  |  | **undiff** | **undiff** | **undiff** |  | **undiff** | **undiff** | **undiff** |  | **1 mon** | **1 mon** | **3 month** | **4 month** |
| BTK | 29.67 | 28.00 | 30.86 |  | **35** | 33.98 | 32.83 |  | 30.48 | 29.66 | 28.27 |  | **35** | **35** | **35** | **35** |
| CASP8 | 30.58 | 29.33 | 31.18 |  | 28.24 | 26.46 | 29.39 |  | 30.54 | 29.49 | 28.40 |  | 29.66 | 31.58 | 28.35 | 29.14 |
| CCL2 | 25.10 | 23.58 | 19.14 |  | 22.00 | 21.06 | 23.11 |  | 24.69 | 23.55 | 22.44 |  | 21.11 | 21.13 | 19.98 | 20.10 |
| CD14 | 32.02 | 30.90 | 29.12 |  | 32.47 | 28.84 | 29.48 |  | 34.65 | 29.23 | 27.18 |  | 26.42 | 27.74 | 27.05 | 26.71 |
| CD80 | 34.98 | **35** | **35** |  | 32.22 | 30.72 | 32.64 |  | 30.33 | 31.37 | 30.36 |  | 34.28 | **35** | **35** | 34.21 |
| CD86 | **35** | **35** | **35** |  | 30.66 | 28.23 | 30.91 |  | 31.16 | 29.43 | 30.59 |  | **35** | **35** | 32.64 | 32.30 |
| CHUK | 25.40 | 24.15 | 25.71 |  | 24.56 | 23.22 | 25.10 |  | 23.46 | 23.17 | 23.43 |  | 25.14 | 27.16 | 24.55 | 25.06 |
| CLEC4E | 34.68 | 33.79 | **35** |  | 30.03 | 29.02 | 33.47 |  | **35** | 30.63 | **35** |  | **35** | **35** | **35** | 34.32 |
| CSF2 | 34.04 | 31.86 | 30.86 |  | 31.59 | 29.19 | 31.58 |  | **35** | **35** | **35** |  | **35** | **35** | 30.86 | 32.10 |
| CSF3 | 29.61 | 28.01 | 30.11 |  | 29.24 | 30.08 | 34.97 |  | **35** | **35** | 34.05 |  | **35** | **35** | **35** | **35** |
| CXCL10 | **35** | 33.28 | 31.15 |  | 34.48 | 31.32 | 33.11 |  | **35** | **35** | 32.42 |  | 31.23 | 31.80 | 29.06 | 28.22 |
| EIF2AK2 | 25.84 | 25.31 | 24.89 |  | 25.40 | 22.11 | 23.93 |  | 24.82 | 23.86 | 24.68 |  | 23.78 | 26.66 | 24.23 | 24.47 |
| ELK1 | 27.02 | 25.68 | 25.47 |  | **35** | 23.58 | 24.51 |  | 24.21 | 23.61 | 22.84 |  | 25.81 | 26.84 | 25.05 | 25.48 |
| FADD | 24.34 | 24.00 | 25.29 |  | 24.46 | 22.37 | 23.78 |  | 23.00 | 22.71 | 22.67 |  | 24.92 | 25.86 | 24.33 | 24.33 |
| FOS | 26.74 | 24.08 | 27.22 |  | 25.73 | 24.01 | 25.31 |  | 25.45 | 24.72 | 24.09 |  | 19.69 | 21.92 | 21.07 | 18.22 |
| HMGB1 | 27.60 | 26.51 | 26.96 |  | 25.71 | 23.84 | 25.21 |  | 24.33 | 24.20 | 25.01 |  | 26.18 | 28.41 | 26.79 | 26.18 |
| HRAS | 23.03 | 21.83 | 22.59 |  | 23.39 | 21.62 | 22.56 |  | 21.47 | 21.18 | 21.82 |  | 23.52 | 25.28 | 23.15 | 23.28 |
| HSPA1A | 23.90 | 23.42 | 24.75 |  | 24.10 | 22.80 | 25.42 |  | 22.06 | 22.71 | 21.68 |  | 23.83 | 24.20 | 22.72 | 23.18 |
| HSPD1 | 22.69 | 21.74 | 23.23 |  | 19.88 | 18.31 | 19.42 |  | 18.25 | 18.29 | 18.26 |  | 21.60 | 23.48 | 21.18 | 21.03 |
| IFNA1 | 30.43 | 29.25 | 30.13 |  | 32.99 | 31.61 | 31.91 |  | 33.73 | 34.07 | 31.43 |  | 33.47 | 31.09 | 30.07 | 28.93 |
| IFNB1 | **35** | **35** | 34.08 |  | **35** | 34.52 | 34.84 |  | **35** | **35** | **35** |  | **35** | 34.97 | 34.61 | **35** |
| IFNG | **35** | **35** | **35** |  | **35** | **35** | **35** |  | **35** | 34.72 | 30.96 |  | **35** | **35** | 34.07 | **35** |
| IKBKB | 27.02 | 25.27 | 25.59 |  | 25.70 | 23.58 | 25.30 |  | 25.80 | 25.24 | 25.27 |  | 26.62 | 28.49 | 25.70 | 25.91 |
| IL10 | **35** | **35** | **35** |  | **35** | **35** | **35** |  | **35** | **35** | **35** |  | **35** | **35** | **35** | **35** |
| IL12A | 30.68 | 29.20 | 29.18 |  | 29.37 | 27.84 | 27.77 |  | 28.28 | 29.36 | 27.61 |  | 30.47 | 32.01 | 29.22 | 28.34 |
| IL1A | **35** | 26.42 | 27.23 |  | 24.63 | 22.75 | 25.50 |  | 26.63 | 27.13 | 26.57 |  | 29.27 | 30.54 | 25.95 | 25.98 |
| IL1B | 32.44 | 33.64 | **35** |  | 23.12 | 24.74 | 26.83 |  | **35** | 31.10 | 27.38 |  | 30.70 | 31.16 | 26.21 | 25.84 |
| IL2 | **35** | **35** | **35** |  | **35** | **35** | **35** |  | 32.99 | 34.23 | **35** |  | **35** | **35** | **35** | 32.63 |
| IL6 | 26.02 | 24.24 | 24.53 |  | 26.65 | 24.29 | 26.78 |  | 28.41 | 29.02 | 26.33 |  | 29.17 | 29.69 | 24.77 | 23.50 |
| IL8 | 22.95 | 22.06 | 22.26 |  | 21.00 | 20.85 | 23.43 |  | 31.19 | 32.82 | 25.23 |  | 26.10 | 25.77 | 22.21 | 21.44 |
| IRAK1 | 24.69 | 25.12 | 24.63 |  | 27.18 | 23.49 | 23.50 |  | 25.79 | 26.65 | 24.80 |  | 25.69 | 25.00 | 23.28 | 24.16 |
| IRAK2 | 26.94 | 25.60 | 25.19 |  | 26.16 | 25.54 | 28.42 |  | 29.10 | 29.08 | 27.89 |  | 28.51 | 29.67 | 27.78 | 27.01 |
| IRF1 | 27.93 | 27.27 | 25.59 |  | 26.94 | 24.68 | 26.57 |  | 27.28 | 26.48 | 26.44 |  | 26.36 | 27.45 | 26.30 | 24.54 |
| IRF3 | 24.08 | 22.94 | 22.84 |  | 22.84 | 20.50 | 21.71 |  | 21.78 | 21.07 | 21.24 |  | 23.42 | 25.01 | 23.14 | 23.34 |
| JUN | 24.62 | 23.54 | 24.27 |  | 26.00 | 23.07 | 24.09 |  | 26.76 | 27.17 | 25.66 |  | 23.34 | 25.71 | 24.46 | 21.40 |
| LTA | **35** | 32.02 | 33.30 |  | 32.88 | 29.24 | **35** |  | **35** | 34.17 | 33.47 |  | **35** | **35** | 33.14 | 33.58 |
| CD180 | 34.80 | 33.93 | **35** |  | **35** | 33.81 | **35** |  | **35** | **35** | 32.94 |  | **35** | **35** | 33.94 | 33.06 |
| LY86 | **35** | **35** | **35** |  | **35** | 34.02 | **35** |  | **35** | **35** | **35** |  | 34.50 | **35** | 33.55 | 33.22 |
| LY96 | 25.49 | 24.06 | 23.51 |  | 30.35 | 28.21 | 29.70 |  | 32.06 | 31.04 | 29.43 |  | 27.26 | 27.62 | 25.69 | 27.22 |
| MAP2K3 | 25.42 | 24.28 | 24.54 |  | 24.99 | 23.06 | 23.46 |  | 23.29 | 23.50 | 23.32 |  | 24.92 | 25.62 | 24.13 | 24.61 |
| MAP2K4 | 26.38 | 25.18 | 25.06 |  | 24.49 | 22.71 | 24.02 |  | 23.18 | 23.09 | 23.09 |  | 24.15 | 25.24 | 23.88 | 24.43 |
| MAP3K1 | 27.15 | 26.15 | 26.42 |  | 24.40 | 22.72 | 24.49 |  | 23.37 | 23.19 | 22.72 |  | 24.49 | 26.63 | 25.69 | 25.95 |
| MAP3K7 | 26.16 | 25.22 | 24.41 |  | 24.74 | 22.46 | 23.25 |  | 23.39 | 23.09 | 23.21 |  | 24.55 | 25.81 | 24.37 | 24.83 |
| MAP3K7IP1 | 25.56 | 25.15 | 24.43 |  | 26.01 | 22.76 | 23.35 |  | 23.69 | 23.32 | 23.34 |  | 24.84 | 26.28 | 24.26 | 24.46 |
| MAP4K4 | 22.18 | 21.30 | 21.54 |  | 23.14 | 21.22 | 22.04 |  | 21.52 | 21.62 | 21.63 |  | 23.44 | 24.99 | 22.44 | 23.03 |
| MAPK8 | 25.03 | 24.10 | 23.87 |  | 24.39 | 21.87 | 22.62 |  | 22.58 | 22.37 | 22.52 |  | 23.57 | 25.51 | 23.59 | 24.20 |
| MAPK8IP3 | 25.77 | 25.38 | 25.11 |  | 26.28 | 23.08 | 24.49 |  | 25.03 | 24.68 | 25.11 |  | 24.54 | 26.35 | 24.59 | 24.12 |
| MYD88 | 25.63 | 24.43 | 24.96 |  | 26.59 | 24.34 | 25.17 |  | 26.17 | 26.00 | 25.83 |  | 24.82 | 27.89 | 25.59 | 25.85 |
| NFKB1 | 24.60 | 23.23 | 23.01 |  | 23.90 | 22.50 | 23.93 |  | 24.65 | 24.42 | 24.10 |  | 24.10 | 24.86 | 23.97 | 23.99 |
| NFKB2 | 29.04 | 28.06 | 27.99 |  | 29.15 | 26.34 | 27.22 |  | 28.04 | 26.57 | 26.99 |  | 27.69 | 28.74 | 27.39 | 26.88 |
| NFKBIA | 24.64 | 23.45 | 22.82 |  | 21.89 | 20.91 | 22.51 |  | 22.03 | 21.94 | 22.07 |  | 23.35 | 24.68 | 22.88 | 21.96 |
| NFKBIL1 | 27.15 | 26.94 | 26.24 |  | 27.67 | 24.13 | 25.39 |  | 26.41 | 25.79 | 25.90 |  | 26.84 | 28.22 | 25.96 | 25.69 |
| NFRKB | 25.82 | 25.13 | 25.00 |  | 26.17 | 23.34 | 23.95 |  | 23.70 | 23.52 | 23.82 |  | 25.19 | 27.12 | 25.47 | 24.94 |
| NR2C2 | 25.89 | 25.16 | 24.29 |  | 25.03 | 22.80 | 24.24 |  | 23.44 | 23.05 | 23.43 |  | 24.47 | 26.40 | 24.32 | 25.07 |
| PELI1 | 25.47 | 24.51 | 23.86 |  | 23.71 | 21.30 | 22.35 |  | 22.05 | 22.07 | 22.17 |  | 24.67 | 26.97 | 25.49 | 24.66 |
| PPARA | 27.26 | 26.62 | 25.17 |  | 27.29 | 24.38 | 25.83 |  | 25.93 | 25.46 | 24.54 |  | 25.33 | 26.85 | 25.41 | 25.02 |
| PRKRA | 26.01 | 24.66 | 24.84 |  | 24.01 | 22.33 | 24.12 |  | 22.96 | 22.24 | 22.75 |  | 24.47 | 26.62 | 25.08 | 24.99 |
| PTGS2 | 25.18 | 23.46 | 24.26 |  | 26.73 | 25.43 | 27.86 |  | 29.24 | 29.25 | 28.68 |  | 27.78 | 28.87 | 24.26 | 22.43 |
| REL | 27.25 | 26.08 | 25.84 |  | 25.46 | 23.14 | 25.03 |  | 25.46 | 25.02 | 25.28 |  | 26.34 | 28.20 | 26.78 | 25.26 |
| RELA | 23.51 | 22.83 | 23.31 |  | 25.75 | 23.58 | 24.76 |  | 26.17 | 25.56 | 25.43 |  | 23.36 | 25.48 | 24.06 | 23.98 |
| RIPK2 | 25.22 | 24.20 | 24.04 |  | 24.44 | 22.10 | 23.16 |  | 23.06 | 23.34 | 22.96 |  | 24.88 | 26.45 | 24.07 | 23.80 |
| SARM1 | 26.95 | 26.58 | 27.15 |  | 28.64 | 25.65 | 26.82 |  | 25.93 | 25.40 | 25.76 |  | 24.85 | 26.35 | 25.25 | 24.55 |
| SIGIRR | 28.88 | 28.16 | 29.08 |  | 32.11 | 27.38 | 29.83 |  | 28.26 | 30.73 | 27.82 |  | 32.14 | 34.53 | 34.15 | 31.89 |
| ECSIT | 25.41 | 24.77 | 24.15 |  | 25.25 | 22.63 | 23.27 |  | 23.05 | 23.47 | 23.26 |  | 24.81 | 26.35 | 24.22 | 23.91 |
| TBK1 | **35** | 24.70 | 25.02 |  | 25.27 | 23.46 | 24.59 |  | 23.94 | 23.71 | 24.06 |  | 25.54 | 27.04 | 25.03 | 25.95 |
| TICAM2 | 27.34 | 26.49 | 26.00 |  | 27.86 | 25.85 | 27.88 |  | 27.81 | 26.55 | 26.11 |  | 27.08 | 27.86 | 26.10 | 25.88 |
| TIRAP | 28.22 | 27.60 | 27.33 |  | 28.53 | 25.46 | 26.35 |  | 26.42 | 26.52 | 27.25 |  | 27.41 | 28.85 | 27.24 | 27.18 |
| TLR1 | 28.93 | 28.14 | 30.46 |  | 33.22 | 33.30 | **35** |  | **35** | **35** | 31.48 |  | 33.95 | **35** | 31.47 | 30.93 |
| TLR10 | **35** | 32.59 | **35** |  | **35** | **35** | **35** |  | 33.38 | **35** | **35** |  | 34.88 | **35** | 32.57 | **35** |
| TLR2 | **35** | **35** | 31.99 |  | 28.53 | 26.99 | 29.45 |  | 29.13 | 29.74 | 28.37 |  | 30.31 | 31.19 | 27.59 | 27.99 |
| TLR3 | 30.36 | 29.25 | 28.18 |  | 30.08 | 27.75 | 29.87 |  | 29.62 | 29.42 | 28.80 |  | 28.42 | 30.08 | 26.78 | 26.48 |
| TLR4 | 25.34 | 24.13 | 23.97 |  | 27.79 | 27.18 | 28.43 |  | 31.16 | 30.12 | 29.10 |  | 27.06 | 30.22 | 26.52 | 27.00 |
| TLR5 | 33.04 | 33.06 | 31.44 |  | 30.54 | 27.65 | 30.35 |  | 30.66 | 29.43 | 29.67 |  | 31.71 | 33.36 | 27.37 | 27.80 |
| TLR6 | 28.27 | 27.22 | 29.03 |  | 29.94 | 28.24 | 30.09 |  | 33.65 | 30.41 | 28.04 |  | 28.63 | 29.30 | 28.06 | 28.04 |
| TLR7 | **35** | **35** | **35** |  | **35** | 31.70 | 33.52 |  | 34.35 | 32.67 | 32.42 |  | 34.30 | **35** | 30.59 | 31.04 |
| TLR8 | **35** | **35** | **35** |  | **35** | 34.44 | 34.61 |  | **35** | **35** | **35** |  | **35** | **35** | **35** | **35** |
| TLR9 | **35** | 34.09 | **35** |  | 33.57 | 32.61 | 33.12 |  | **35** | 32.61 | 34.73 |  | 32.70 | **35** | 33.18 | 33.20 |
| TNF | **35** | **35** | 32.04 |  | 28.44 | 25.41 | 30.94 |  | **35** | 32.33 | 29.37 |  | 31.52 | 32.47 | 31.56 | 31.88 |
| TNFRSF1A | 27.23 | 26.16 | 26.39 |  | 27.23 | 24.78 | 26.03 |  | 26.54 | 26.17 | 25.17 |  | 26.41 | 27.22 | 25.01 | 25.73 |
| TOLLIP | 26.28 | 25.10 | 25.33 |  | 25.70 | 24.12 | 24.91 |  | 24.83 | 24.68 | 24.67 |  | 25.79 | 26.57 | 23.25 | 25.04 |
| TRAF6 | 25.96 | 25.17 | 26.04 |  | 25.82 | 24.35 | 25.74 |  | 24.54 | 25.57 | 24.78 |  | 25.52 | 26.73 | 24.33 | 25.01 |
| TICAM1 | 25.44 | 24.10 | 24.08 |  | 26.94 | 25.86 | 27.24 |  | 28.10 | 27.53 | 26.03 |  | 26.20 | 26.57 | 24.18 | 25.07 |
| UBE2N | 23.19 | 22.41 | 22.07 |  | 22.53 | 20.53 | 21.08 |  | 20.94 | 20.80 | 20.38 |  | 22.80 | 23.26 | 21.62 | 22.75 |
| UBE2V1 | 29.96 | 28.49 | 33.23 |  | 28.20 | 25.60 | 27.06 |  | 26.72 | 26.46 | 26.64 |  | 28.72 | 31.19 | 28.31 | 29.58 |
|  |  |  |  |  |  |  |  |  |  |  |  |  |  |  |  |  |
|  |  |  |  |  |  |  |  |  |  |  |  |  |  |  |  |  |
|  |  |  |  |  |  |  |  |  |  |  |  |  |  |  |  |  |
| housekeeping | |  |  |  |  |  |  |  |  |  |  |  |  |  |  |  |
| B2M | 20.8 | 19.72 | 19.45 |  | 20.25 | 18.47 | 20.11 |  | 22.43 | 22.04 | 21.19 |  | 20.32 | 20.89 | 17.92 | 19.03 |
| HPRT1 | 24.58 | 23.55 | 23.59 |  | 22.9 | 21.03 | 22.11 |  | 22.16 | 22.47 | 21.28 |  | 23.75 | 24.3 | 22.21 | 22.86 |
| RPL13A | 20.72 | 19.59 | 19.36 |  | 19.82 | 17.89 | 19.22 |  | 18.97 | 19.14 | 19.09 |  | 20.51 | 21.16 | 18.29 | 19.45 |
| GAPDH | 18.58 | 17.04 | 17.39 |  | 17.26 | 16.04 | 17.06 |  | 16.37 | 16.41 | 16.8 |  | 18.42 | 20.81 | 16.19 | 17.13 |
| ACTB | 17.97 | 17.15 | 18.68 |  | 16.95 | 15.81 | 17.36 |  | 16.24 | 16.56 | 16.14 |  | 18.04 | 18.72 | 15.19 | 17.21 |
| ave HKPG | **20.53** | **19.41** | **19.69** |  | **19.44** | **17.84** | **19.17** |  | **19.23** | **19.32** | **18.9** |  | **20.2** | **21.17** | **17.96** | **19.13** |
