# Supplementary material for: Innate Immunity in Human Embryonic Stem Cells: Comparison with Adult Human Endothelial Cells
Source: PLoS One. 2010 May 5;5(5):e10501. doi: 10.1371/journal.pone.0010501 (PMC2864770; doi:10.1371/journal.pone.0010501)
Supplement: Table S2 — Fold changes between undifferentiated H7 hESC (n = 3) and differentiated H7 at 1 month (n = 2), 3 months and 4 months after differentiation. N.d.d. = not detectable in differentiated; n.d.u. = not detectable in 3/3 or 2/3 undifferentiated and n.d. = not detectable in either. (0.14 MB DOC) [file pone.0010501.s003.doc]

| **Table S2** | **Differentiated H7: fold change vs undifferentiated** | | | |
| --- | --- | --- | --- | --- |
| **Gene Name** | 1 month | 1 month | 3 month | 4 month |
| BTK | **n.d.d** | **n.d.d** | **n.d.d** | **n.d.d** |
| CASP8 | 0.85 | 0.44 | 0.56 | 0.58 |
| CCL2 | 5.04 | 9.73 | 2.33 | 4.84 |
| CD14 | 37.55 | 29.44 | 5.12 | 14.63 |
| CD80 | 0.49 | **n.d.d** | **n.d.d** | 0.24 |
| CD86 | **n.d.d** | **n.d.d** | 0.08 | 0.24 |
| CHUK | 1.45 | 0.70 | 0.46 | 0.73 |
| CLEC4E | **n.d.d** | **n.d.d** | **n.d.d** | 0.11 |
| CSF2 | **n.d.d** | **n.d.d** | 0.52 | 0.50 |
| CSF3 | **n.d.d** | **n.d.d** | **n.d.d** | **n.d.d** |
| CXCL10 | 8.74 | 11.52 | 8.29 | 33.54 |
| EIF2AK2 | 2.68 | 0.71 | 0.41 | 0.79 |
| ELK1 | 0.00 | 0.00 | 0.00 | 0.00 |
| FADD | 1.00 | 1.02 | 0.32 | 0.72 |
| FOS | 104.97 | 43.80 | 8.51 | 138.55 |
| HMGB1 | 1.09 | 0.46 | 0.15 | 0.52 |
| HRAS | 1.31 | 0.76 | 0.36 | 0.74 |
| HSPA1A | 3.17 | 4.80 | 1.44 | 2.37 |
| HSPD1 | 0.50 | 0.26 | 0.14 | 0.35 |
| IFNA1 | 1.06 | 10.82 | 2.37 | 11.77 |
| IFNB1 | **n.d.d** | 0.00 | 0.00 | **n.d.d** |
| IFNG | **n.d** | **n.d** | **n.d.u** | **n.d** |
| IKBKB | 0.77 | 0.41 | 0.31 | 0.60 |
| IL10 | **n.d** | **n.d** | **n.d** | **n.d** |
| IL12A | 0.59 | 0.40 | 0.30 | 1.23 |
| IL1A | 0.08 | 0.07 | 0.17 | 0.39 |
| IL1B | 0.05 | 0.07 | 0.22 | 0.65 |
| IL2 | **n.d.u** | **n.d.u** | **n.d.u** | **n.d.u** |
| IL6 | 0.27 | 0.37 | 1.21 | 6.61 |
| IL8 | 0.13 | 0.32 | 0.40 | 1.56 |
| IRAK1 | 1.34 | 4.23 | 1.50 | 1.84 |
| IRAK2 | 0.75 | 0.66 | 0.26 | 1.01 |
| IRF1 | 2.13 | 1.96 | 0.47 | 3.58 |
| IRF3 | 0.78 | 0.51 | 0.20 | 0.40 |
| JUN | 5.40 | 2.05 | 0.52 | 9.88 |
| LTA | **n.d.d** | **n.d.d** | 0.00 | 0.00 |
| CD180 | **n.d.u** | **n.d.u** | **n.d.u** | **n.d.u** |
| LY86 | **n.d.u** | **n.d.u** | **n.d.u** | **n.d.u** |
| LY96 | 10.78 | 16.45 | 6.75 | 5.28 |
| MAP2K3 | 1.23 | 1.49 | 0.45 | 0.73 |
| MAP2K4 | 1.97 | 1.81 | 0.50 | 0.77 |
| MAP3K1 | 1.70 | 0.76 | 0.16 | 0.29 |
| MAP3K7 | 1.25 | 1.02 | 0.30 | 0.49 |
| MAP3K7IP1 | 1.39 | 1.00 | 0.44 | 0.86 |
| MAP4K4 | 1.06 | 0.71 | 0.45 | 0.67 |
| MAPK8 | 1.71 | 0.87 | 0.36 | 0.53 |
| MAPK8IP3 | 2.76 | 1.96 | 0.56 | 1.76 |
| MYD88 | 3.82 | 0.89 | 0.47 | 0.89 |
| NFKB1 | 1.66 | 1.92 | 0.38 | 0.85 |
| NFKB2 | 2.41 | 2.28 | 0.63 | 2.01 |
| NFKBIA | 1.12 | 0.68 | 0.26 | 1.09 |
| NFKBIL1 | 1.21 | 0.91 | 0.47 | 1.28 |
| NFRKB | 1.61 | 0.83 | 0.28 | 0.91 |
| NR2C2 | 1.92 | 0.99 | 0.45 | 0.60 |
| PELI1 | 0.52 | 0.21 | 0.06 | 0.25 |
| PPARA | 3.71 | 2.53 | 0.74 | 2.19 |
| PRKRA | 1.32 | 0.58 | 0.18 | 0.44 |
| PTGS2 | 1.21 | 1.12 | 2.94 | 23.60 |
| REL | 0.75 | 0.41 | 0.12 | 0.76 |
| RELA | 6.61 | 2.98 | 0.86 | 2.05 |
| RIPK2 | 0.84 | 0.55 | 0.31 | 0.84 |
| SARM1 | 11.91 | 10.50 | 1.90 | 6.99 |
| SIGIRR | 0.51 | 0.19 | 0.03 | 0.29 |
| ECSIT | 1.23 | 1.05 | 0.39 | 1.09 |
| TBK1 | 1.22 | 0.84 | 0.37 | 0.44 |
| TICAM2 | 2.84 | 3.23 | 1.18 | 3.10 |
| TIRAP | 1.56 | 1.12 | 0.37 | 0.87 |
| TLR1 | 0.00 | **n.d.d** | 0.00 | 0.00 |
| TLR10 | **n.d.u** | **n.d.u** | **n.d.u** | **n.d.u** |
| TLR2 | 0.66 | 0.70 | 0.92 | 1.57 |
| TLR3 | 4.60 | 2.85 | 3.02 | 8.40 |
| TLR4 | 4.37 | 0.96 | 1.34 | 2.17 |
| TLR5 | 0.53 | 0.33 | 2.25 | 3.77 |
| TLR6 | 4.53 | 5.58 | 1.42 | 3.25 |
| TLR7 | 0.00 | **n.d.d** | 0.00 | 0.00 |
| TLR8 | **n.d.d** | **n.d.d** | **n.d.d** | **n.d.d** |
| TLR9 | 3.45 | **n.d.d** | 0.52 | 1.16 |
| TNF | 0.27 | 0.28 | 0.06 | 0.10 |
| TNFRSF1A | 1.99 | 2.22 | 1.11 | 1.52 |
| TOLLIP | 1.42 | 1.62 | 1.74 | 1.14 |
| TRAF6 | 2.08 | 1.76 | 1.00 | 1.41 |
| TICAM1 | 3.65 | 5.53 | 3.12 | 3.80 |
| UBE2N | 0.98 | 1.39 | 0.47 | 0.48 |
| UBE2V1 | 0.77 | 0.27 | 0.22 | 0.20 |
